# Structure–Activity Relationships of Quinoxaline-Based 5-HT_3_A and 5-HT_3_AB Receptor-Selective Ligands

**DOI:** 10.1002/cmdc.201300032

**Published:** 2013-05-02

**Authors:** Andrew J Thompson, Mark H P Verheij, Jacqueline E van Muijlwijk-Koezen, Sarah C R Lummis, Rob Leurs, Iwan J P de Esch

**Affiliations:** [a] Amsterdam Institute for Molecules Medicines and Systems (AIMMS), Division of Medicinal Chemistry, Faculty of Sciences, VU University Amsterdam Amsterdam (The Netherlands); [b] Department of Biochemistry, University of Cambridge Cambridge (UK) E-mail: sl120@cam.ac.uk

**Keywords:** Cys loops, heteromers, homomers, 5-HT_3_ receptors, quinoxalines, serotonin, subtypes

## Abstract

Until recently, discriminating between homomeric 5-HT_3_A and heteromeric 5-HT_3_AB receptors was only possible with ligands that bind in the receptor pore. This study describes the first series of ligands that can discriminate between these receptor types at the level of the orthosteric binding site. During a recent fragment screen, 2-chloro-3-(4-methylpiperazin-1-yl)quinoxaline (VUF10166) was identified as a ligand that displays an 83-fold difference in [^3^H]granisetron binding affinity between 5-HT_3_A and 5-HT_3_AB receptors. Fragment hit exploration, initiated from VUF10166 and 3-(4-methylpiperazin-1-yl)quinoxalin-2-ol, resulted in a series of compounds with higher affinity at either 5-HT_3_A or 5-HT_3_AB receptors. These ligands reveal that a single atom is sufficient to change the selectivity profile of a compound. At the extremes of the new compounds were 2-amino-3-(4-methylpiperazin-1-yl)quinoxaline, which showed 11-fold selectivity for the 5-HT_3_A receptor, and 2-(4-methylpiperazin-1-yl)quinoxaline, which showed an 8.3-fold selectivity for the 5-HT_3_AB receptor. These compounds represent novel molecular tools for studying 5-HT_3_ receptor subtypes and could help elucidate their physiological roles.

## Introduction

5-HT_3_ receptors are ligand-gated ion channels that are responsible for fast synaptic neurotransmission in the central (CNS) and peripheral nervous systems (PNS). They are involved in physiological functions as diverse as the vomiting reflex, pain processing, reward, cognition, and anxiety, and modulate the release of neurotransmitters such as acetylcholine, cholecystokinin, dopamine, GABA, glutamate, and serotonin itself.^1^ To date, five different subunits (5-HT3A–5-HT3E) have been identified, but the homomeric 5-HT_3_A- and heteromeric 5-HT_3_AB-containing receptors are the most fully characterized.^1^, ^2^ 5-HT_3_A receptors are located primarily in the CNS, while 5-HT_3_AB receptors may be more abundant in the PNS.^1^, ^3^

5-HT_3_ receptors are members of the Cys-loop family of neurotransmitter-gated receptors that all share a pentameric structure of subunits surrounding a central ion-conducting pore. Each subunit has an extracellular domain, four transmembrane α helices (one of which contributes to the ion conducting pore) and an intracellular domain.^4^ The agonist/competitive antagonist (orthosteric) binding site is located at the interface of two adjacent subunits and is formed by the convergence of three loops (loops A–C) from the principal (or +) subunit and three β sheets (loops D–E) from the adjacent complementary (or −) subunit.

**Figure 1:**
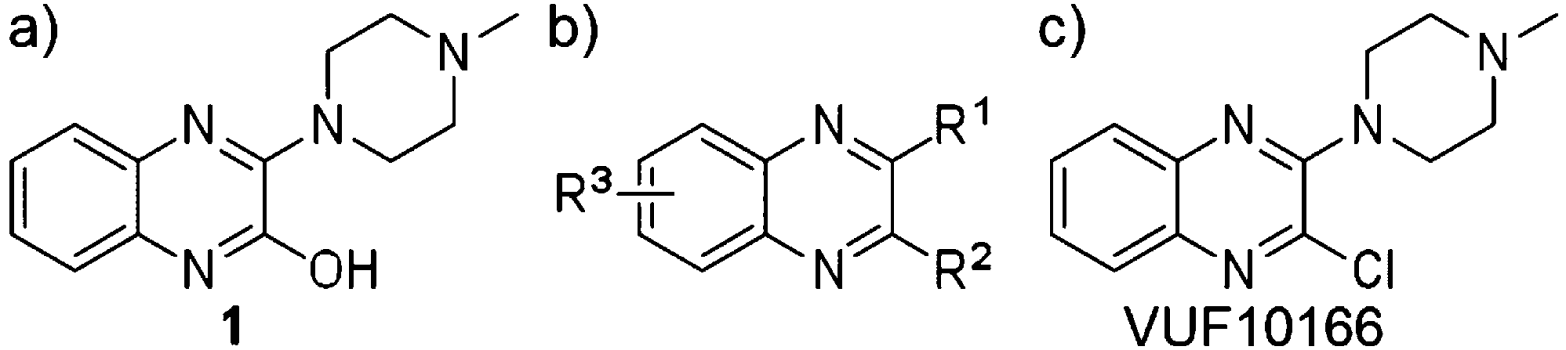
a) Structure of compound **1**; b) general structure for all analogues; c) VUF10166.

The two 5-HT_3_ receptor subtypes (5-HT_3_A and 5-HT_3_AB) can be distinguished by differences in their 5-HT concentration–response curves (increased EC_50_ values and shallower Hill slopes), increased single channel conductance (5-HT_3_A=sub-pS; 5-HT_3_AB=16-30 pS), increased rate of desensitization, decreased relative Ca^2+^ permeability, and different current–voltage relationships (5-HT_3_A is inwardly rectifying, 5-HT_3_AB is linear).1b, 5 Pharmacologically distinguishing 5-HT_3_A from 5-HT_3_AB receptors has historically required the use of compounds that bind in the pore, such as bilobalide, ginkgolide, and picrotoxinin.^6^ In contrast, competitive ligands usually have very similar affinities at 5-HT_3_A and 5-HT_3_AB receptors. Recently, however, a quinoxaline compound (VUF10166) was identified that showed differences in both its binding affinity and functional properties at 5-HT_3_A and 5-HT_3_AB receptors (Figure 1).^7^ Detailed studies of VUF10166 showed that these differences may stem from a second, allosteric, site only found in the 5-HT_3_AB receptor, the occupation of which may increase the rate of ligand dissociation from the adjacent orthosteric site.

The actions of a range of quinoxalines have also been previously studied at both 5-HT_3_A and native receptors and revealed that these compounds can be relatively potent (sub- micromolar affinities) as antagonists, agonists, and partial agonists, with potential as novel therapeutics.^8^ There is particular interest, for example, in developing quinoxalines which are impermeable to the blood–brain barrier that would target peripheral 5-HT_3_ receptors.8a None of these studies, however, have evaluated ligand affinities at specific 5-HT_3_ receptor subtypes. In this manuscript, we report the synthesis and binding affinities of a series of quinoxalines and demonstrate subtle differences in structure–activity relationships (SAR) for the 5-HT_3_A and 5-HT_3_AB receptor subtypes using competition binding on recombinantly expressed receptors in HEK293 cells.

## Results and Discussion

### Chemistry

**Scheme 1:**
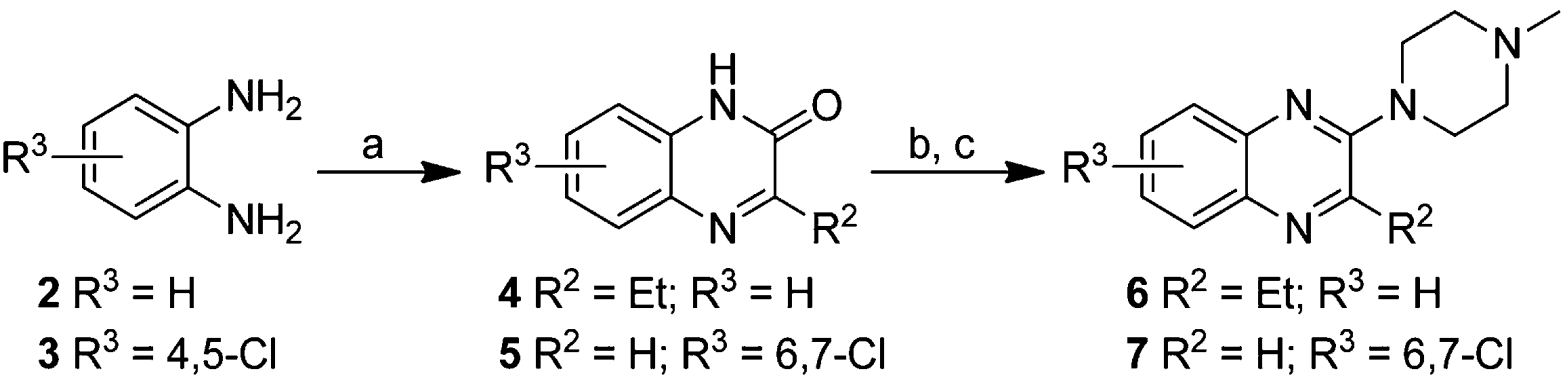
Synthesis of quinoxalines. *Reagents and conditions*: a) R_2_COCO_2_H, CH_3_OH, RT, 30 min; b) POCl_3_, 100 °C, 1 h; c) *N*-methylpiperazine, mw, 120 °C, or *N*-methylpiperazine, EtOAc, mw, 160 °C, 15 min.

The pharmacophore features of lead compound **1** and VUF10166, and the effects of these features on 5-HT_3_A and 5-HT_3_AB receptor affinities, was explored by screening a series of compounds that contain the quinoxaline scaffold (Figure 1 b). Intermediates **4**–**5** were synthesized via a two-step ring formation between 2-amino aniline **2** or **3** and the appropriate 2-oxo carboxylic acids (Scheme 1). After conversion into the corresponding 2-chloroquinoxalines with phosphorylchloride, subsequent nucleophilic aromatic substitution with *N*-methylpiperazine under microwave conditions gave compounds **6** and **7** in moderate to good yields.

**Scheme 2:**
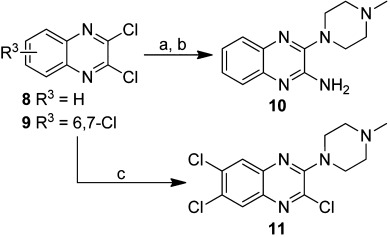
Synthesis of compounds **10** and **11**. *Reagents and conditions*: a) 2 m NH_3_ in EtOH, mw, 100 °C, 120 min; b) *N*-methylpiperazine, THF, mw, 150 °C, 40 min; c) *N*-methylpiperazine, Et_3_N, THF, 80 °C, 96 h.

Starting from commercially available chloro-quinoxalines **8** and **9**, different synthetic routes were followed to synthesize compounds **10** and **11** (Scheme 2). Compound **10** was synthesized through two subsequent nucleophilic aromatic substitution reactions. First, the amine moiety was introduced by reacting compound **8** with ammonia in ethanol. Subsequently, the *N*-methylpiperazine group was introduced. Both reactions were performed under microwave conditions. Compound **11** was created in a similar manner, although conventional heating was used for this synthesis.

**Scheme 3:**
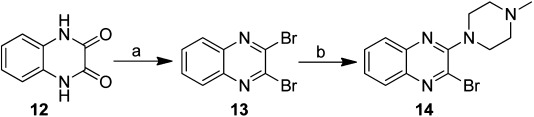
Synthesis of 2-bromo-3-(4-methylpiperazin-1-yl)quinoxaline (**14**). *Reagents and conditions*: a) PBr_5_, toluene, 160 °C, 3 h; b) *N*-methylpiperazine, Et_3_N, toluene, reflux, 6 h.

**Scheme 4:**
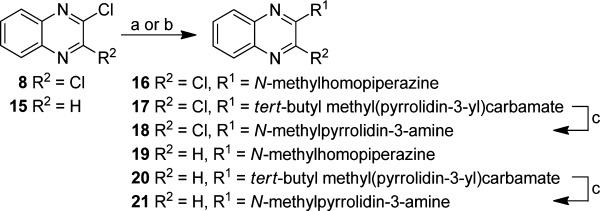
Synthesis of compounds **16**, **18**, **19**, and **21**. *Reagents and conditions*: a) *N*-methylhomopiperazine, Et_3_N, toluene, reflux, overnight; b) *tert*-butyl methyl(pyrrolidin-3-yl)carbamate, K_2_CO_3_, DMF, 90 °C, 4 h; c) 4 m HCl in dioxane, RT, overnight.

**Scheme 5:**
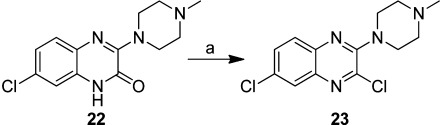
Synthesis of compound **23**. *Reagents and conditions*: a) POCl_3_, DiPEA, toluene, reflux, 20 h.

Commercially available quinoxaline-2,3(1*H*,4*H*)-dione (**12**) was treated with phosphorous pentabromide to form 2,3-dibromoquinoxaline (**13**), which was then allowed to react with *N*-methylpiperazine in toluene at reflux to yield compound **14** (Scheme 3). For compounds **16**, **18**, **19**, and **21**, 2,3-dichloroquinoxaline (**8**) or 2-chloroquinoxaline (**15**) were reacted with the corresponding amines using various solvents and temperatures to yield **16**, **17**, **19**, and **20** in good yields. Boc-protected intermediates **17** and **20** were subsequently deprotected with a 4 m solution of hydrochloric acid in dioxane to give compounds **18** and **21** (Scheme 4). The regioselective synthesis of compound **22** was described earlier by our group.^9^ Here, we used this compound as a precursor in the synthesis of compound **23** (Scheme 5).

### Biochemical evaluation and SAR studies

#### SAR of quinoxaline compounds for the 5-HT_3_A receptors

Target compounds were evaluated using competition binding with the 5-HT_3_-specific ligand [^3^H]granisetron; the results are summarized in Table 1. SAR data in this table are presented with a focus on different substitution patterns at the R^1^, R^2^, and R^3^ positions of the quinoxaline core scaffold. We found that several quinoxaline compounds show clear differences in their binding affinities at the two receptor subtypes, and the subtype preference differs within the series.

**Table 1:** Competition binding affinities for quinoxalines at 5-HT_3_A and 5-HT_3_AB receptors with respect to substitutions at R^1^, R^2^, and R^3^

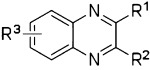								
Compd	R^1^	R^2^	R^3^	p*K*_i_ (A)	*n*	p*K*_i_ (AB)	*n*	Fold diff.^[a]^
**1**	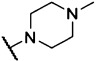	OH	H	8.93±0.21	11	9.36±0.06	11	+2.7
**28**	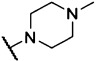	OMe	H	9.18±0.16	6	8.34±0.10	7	−7.1
**29**	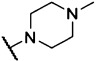	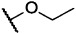	H	9.00±0.22	7	8.24±0.10	7	−5.8
**30**	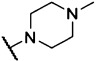	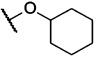	H	6.27±0.20	4	6.71±0.30	4	+2.8
**31**	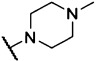	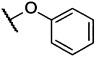	H	7.06±0.07	2	6.77±0.39	2	−2.0
**32**	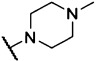	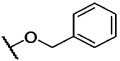	H	6.89±0.12	4	7.12±0.13	4	+1.7
**10**	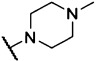	NH_2_	H	8.53±0.11	5	7.51±0.32	5	−11
**26**	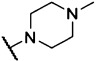	Me	H	8.59±0.19	5	8.42±0.09	5	−1.5
**6**	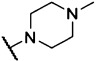	Et	H	9.20±0.12	5	8.84±0.46	8	−2.3
**27**	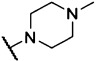	CF_3_	H	7.35±0.15	3	7.42±0.39	3	+1.2
**14**	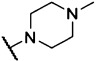	Br	H	9.31±0.16	6	8.85±0.16	7	−2.9
VUF10166	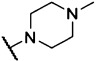	Cl	H	9.82±0.26	7	7.90±0.49	6	−83
**16**	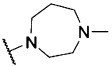	Cl	H	9.11±0.26	3	8.34±0.23	3	−5.9
**18**	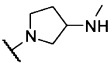	Cl	H	6.69±0.16	3	6.67±0.02	3	−1.0
**23**	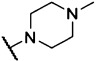	Cl	6-Cl	8.95±0.18	7	7.85±0.08	14	−13
**11**	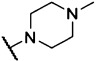	Cl	6,7-Cl	8.09±0.34	6	7.48±0.16	6	−4.1
**22**	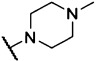	OH	7-Cl	7.50±0.13	3	8.14±0.21	3	+4.3
**34**	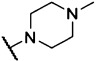	OH	6,7-Cl	7.30±0.39	6	7.14±0.26	9	−1.4
**24**	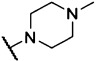	H	H	8.21±0.26	5	9.13±0.30	5	+8.3
**33**	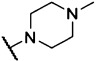	H	6-Cl	7.79±0.20	3	6.99±0.31	3	−6.3
**7**	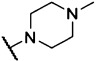	H	6,7-Cl	8.41±0.29	6	8.07±0.27	5	−2.2
**19**	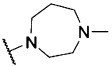	H	H	8.09±0.20	5	7.48±0.13	5	−4.0
**21**	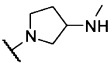	H	H	7.49±0.08	6	7.11±0.15	5	−2.4

[a]+/− refer to an increase or decrease at 5-HT_3_AB relative to 5-HT_3_A.

First, the SAR of the series will be described for the 5-HT_3_A receptor subtype. The alcohol moiety at the R^2^ position implies that compound **1** can adopt two different tautomeric states. It seems that the tautomeric form in which the aromatic nitrogen atom represents a hydrogen bond donor is not involved in binding, as the conversion of the R^2^ alcohol functionality of **1** into a methoxy (compound **28**) or ethoxy (compound **29**) group results in compounds with comparable affinities. However, larger ether analogues are not favorable for binding, as observed for compounds **30**–**32** in which the cyclohexyl, phenyl, and benzyl ether derivatives have ∼100-fold lower affinities. When the hydroxy group of **1** at R^2^ was changed to a different polar moiety (e.g., an amine moiety, as in compound **10**), the high affinity was maintained. A decreased affinity was observed for compound **26**, which incorporates a methyl group (which is electron-donating) at the R^2^ position, relative to VUF10166. Addition of an electron-withdrawing CF_3_ group (compound **27**) results in an even larger decrease in 5-HT_3_A receptor affinity. Compounds that have chlorine or bromine atoms at this position have sub-nanomolar affinities (VUF10166 and **14**), indicating that the SAR in this position is very subtle, and an interplay between inductive and resonance effects cannot be ruled out.

For R^2^=Cl (VUF10166), different basic moieties were introduced. A small drop in affinity results from replacing R^1^=*N*-methylpiperazine (VUF10166) with R^1^=*N*-methylhomopiperazine (**16**), but a ∼1000-fold drop in affinity is observed for R^1^=*N*-methylpyrrolidin-3-amine (**18**). As the methylpiperazine moiety leads to the most potent compounds at 5-HT_3_A receptors, this basic group was used in the R^1^ position when exploring the effects of different chlorine substitution patterns at the R^3^ position. Addition of a 6-Cl at R^2^ (compound **23**) causes a ∼10-fold drop in affinity, and a second chlorine atom at position R^3^ (6,7-Cl, **11**) results in another ∼10-fold decrease. Again, VUF10166 (R^3^=H) shows the highest affinity for the 5-HT_3_A receptor. For compounds with R^2^=OH (**1**), a similar trend is observed. Affinity at the 5-HT_3_A receptor is highest for R^3^=H (**1**) and decreases significantly for both compound **22** (R^3^=6-Cl) and **34** (R^3^=6,7-Cl), which both have a p*K*_i_ in the mid-nanomolar range.

The same modifications to R^1^ and R^3^ were made for the most simple 2-*N*-methylpiperazine quinoxaline compound of the series (R^2^=H, **24**), which has a p*K*_i_ of 8.21. Addition of chlorine atoms at position R^3^ (**33**, **7**) results in compounds with similar affinity at the 5-HT_3_A receptor. Finally, replacement of the *N*-methylpiperazine group of compound **24** with an *N*-methylhomopiperazine group (**19**) has no effect on 5-HT_3_A receptor affinity, but for R^1^=*N*-methylpyrrolidin-3-amine (**21**), a small decrease in affinity is observed. This is different to what is observed for R^2^=Cl, where basic moieties other than the *N*-methylpiperazine group resulted in more pronounced differences in affinity (e.g., compare **19** and **21** with **16** and **18**, respectively).

#### Affinity differences at 5-HT_3_A and 5-HT_3_AB receptors

The affinity of compound **1** is slightly higher (2.7-fold) for 5-HT_3_AB receptors than for 5-HT_3_A receptors. Methoxy and ethoxy analogues **28** and **29** both show a 10-fold decrease in affinity at 5-HT_3_AB receptors relative to compound **1**; in contrast, these modifications do not result in a change in affinity at the 5-HT_3_A receptor. The larger ether analogues **30**–**32** have p*K*_i_ values of ∼7 for the 5-HT_3_AB receptor, which are similar to their affinities for the homomeric receptor. Replacement of the alcohol moiety with an amine moiety (compound **10**) resulted in a large decrease in affinity (∼70-fold) for 5-HT_3_AB receptors but had little effect on the affinity for 5-HT_3_A receptors. Similar affinities are observed at both the 5-HT_3_A and 5-HT_3_AB receptors for compounds that have methyl and ethyl substituents in the R^2^ position (i.e., **26** and **6**, respectively), as well as for trifluoromethyl derivative **27**. For the halogen-substituted compounds, a different trend is observed. For R^2^=Br, the p*K*_i_ for 5-HT_3_AB receptors is close to 9, which is similar to that for 5-HT_3_A receptors, but the affinity of VUF10166 (R^2^=Cl) is significantly decreased at 5-HT_3_AB receptors, resulting in a ∼100-fold difference relative to 5-HT_3_A receptors. The effect of replacing R^1^=*N*-methylpiperazine (VUF10166) for R^1^=*N*-methylhomopiperazine while R^2^=Cl (**16**) is negligible at 5-HT_3_AB receptors, which is again different to what is observed at 5-HT_3_A receptors. For R^1^=*N*-methylpyrrolidin-3-amine (**18**), a >10-fold decrease in affinity for 5-HT_3_AB receptors is observed. 5-HT_3_AB receptor affinities for compounds with a chlorine atom at position R^2^ (VUF10166, **23**, and **11**) do not change substantially when increasing numbers of chlorine atoms are added at the R^3^ position, although compound **11** has the lowest 5-HT_3_AB receptor affinity of this subset. This is different to what is observed for the 5-HT_3_A receptor affinities of these compounds, where addition of chlorine at R^3^ resulted in a large decrease in affinity. When R^2^=OH, compound **1** (R^3^=H) had the highest 5-HT_3_AB receptor affinity, compound **22** (R^3^=6-Cl) showed a ∼10-fold decrease in affinity, and a further ∼10-fold decrease in affinity was observed for compound **34** (R^3^=6,7-Cl). 5-HT_3_A receptor affinities shown by **22** and **34** are similar. When R^2^=H, the highest 5-HT_3_AB receptor affinity was observed for R^3^=H (**24**), but a ∼100-fold drop in affinity was observed for R^3^=6-Cl (**33**) and only a ∼10-fold drop for R^3^=6,7-Cl (**7**). The effect on 5-HT_3_AB receptor affinity when replacing R^1^=*N*-methylpiperazine (**24**) for R^1^=*N*-methylhomopiperazine (**19**), while R^2^=H, is a ∼45-fold decrease in affinity. For compound **21**, a similar lowering in 5-HT_3_AB receptor affinity is observed. This is in contrast to what is observed for 5-HT_3_A receptors and can primarily be attributed to the sub-nanomolar affinity of compound **24** at 5-HT_3_AB receptors, which is almost 10-fold higher than its 5-HT_3_A receptor affinity.

Table 1 shows that compound **24** shows the highest selectivity for 5-HT_3_AB over 5-HT_3_A receptors (∼10-fold), and VUF 10166 has the highest selectivity for 5-HT_3_A over 5-HT_3_AB receptors (∼100-fold). The difference between these two compounds is solely the atom at position R^2^, R^2^=H for compound **24** and R^2^=Cl for VUF10166. When the hydrogen atom is replaced with a chlorine atom, the 5-HT_3_A receptor affinity increases ∼40-fold, while the affinity for 5-HT_3_AB receptors decreases ∼20-fold. Both of these compounds comprise the *N*-methylpiperazine moiety, which is the preferred basic group for selectivity. For 5-HT_3_A receptor affinity, R^2^=Cl (VUF10166) is superior, with Br (**14**), Et (**6**), OMe (**28**), and OEt (**29**) having similar lower affinities. For the 5-HT_3_AB receptor, an alcohol moiety at position R^2^ (as observed for compound **1**) is preferred, but a hydrogen atom at R^2^ also results in high affinity (compound **24**). At 5-HT_3_AB receptors, R^2^=Br and the smaller alkyl (**26**, **6**) and ether analogues (**28** and **29**) also have high affinities, whereas incorporation of larger ether groups at R^2^ results in decreased affinity. However, for R^2^=Cl (VUF10166) and NH_2_ (**10**), only 5-HT_3_AB receptor affinity is decreased, resulting in 100- and 10-fold selectivity for 5-HT_3_A over 5-HT_3_AB receptors, respectively. Different substitution patterns at the R^3^ position also caused marked changes. For example, when R^2^=Cl, replacement of R^3^=H (VUF10166) with a chlorine atom results in a ∼10-fold (R^3^=6-Cl, **23**) or ∼100-fold (R^3^=6,7-Cl, **11**) decrease in affinity for the 5-HT_3_A receptor, but this replacement does not have a large effect on 5-HT_3_AB receptor affinity. When R^2^=H, the affinity for 5-HT_3_A receptors does not show a large difference upon addition of chlorine atoms to the R^3^ position, but the 5-HT_3_AB receptor affinity changes significantly. It can be concluded that, in either case, the greatest 5-HT_3_ receptor subtype selectivity is achieved for R^1^=*N*-methylpiperazine.

#### 5-HT_3_ receptor binding sites

Orthosteric binding sites in 5-HT_3_AB receptors could theoretically exist at A+A−, A+B−, B+A−, and B+B− interfaces, but the majority of 5-HT_3_ receptor-competitive ligands only bind to an A+A− interface.^4^, ^10^ There is evidence, however, that at least one of the quinoxaline compounds studied here (VUF10166) binds to both an A+A− and an A+B− interface; binding to the A+B− interface may decrease the affinity of ligands binding to the A+A− site by allosterically increasing the rate of ligand dissociation.^7^ Other quinoxalines may similarly bind to both sites; thus, to identify potential interactions, we constructed models of the two interfaces.

**Figure 2:**
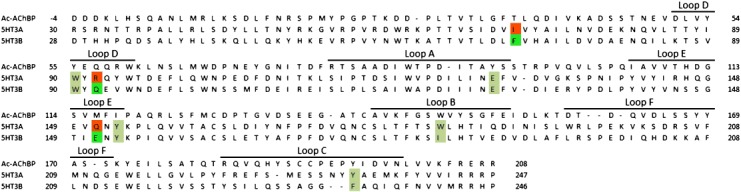
Protein sequence alignment for Ac-AChBP, 5-HT_3_A, and 5-HT_3_B. Residues illustrated in Figure 3 are highlighted. Identical residues are shown in beige, dissimilar residues of the complementary side of the 5-HT_3_A subunit in orange, and residues for the 5-HT_3_B receptor are shown in green. Accession numbers for the Ac-AChBP, 5-HT_3_A and 5-HT_3_B subunits are Q8WSF8, P46098, and O95264, respectively. Note that the numbering of the 5-HT_3_A and 5-HT_3_B residues corresponds to the mouse numbering in order to allow comparison with other work.^11^

**Figure 3:**
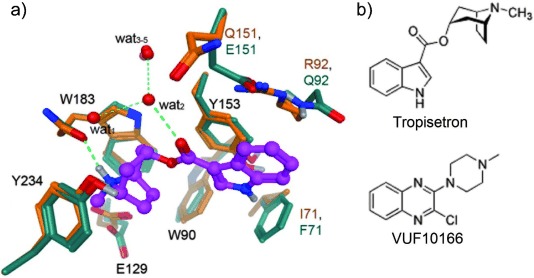
a) Overlay of homology models for the A+A− (protein carbon atoms in orange) and A+B− (protein carbon atoms in green) binding sites containing tropisetron (purple ball and stick) and a network of structural water molecules (oxygen atoms as red balls). Hydrogen bonds for ligand– receptor, ligand–solvent, and solvent–solvent interactions are shown as green dotted lines. Residue annotation for identical residues is in black and, for divergent residues, orange corresponds to 5-HT_3_A subunits and green to 5-HT_3_B subunits. b) Structures of tropisetron and VUF10166 to highlight their similarities.

Homology models were based on a tropisetron-bound AChBP crystal structure (PDB code: 2WNC) as no quinoxaline-bound Cys-loop receptor structure has been solved to date, and tropisetron is the closest structurally related compound to those described here (Figure 3). Tropisetron is an antagonist at the 5-HT_3_ receptor but can act as an agonist at some nACh receptors;^11^ thus, it is an ideal choice from the available structures as quinoxalines can act as both agonists and antagonists at 5-HT_3_ receptors, though they were not evaluated in this study. As with all homology models, caution must be applied in data interpretation, especially now that recent electron microscope images of the nACh receptor have shown that the difference between the structure of unbound and agonist-bound binding site sites is less than that observed in AChBP.^12^ Nevertheless, it is likely that our compounds adopt a broadly similar orientation to tropisetron; therefore, the models serve as means of identifying residues that could potentially be responsible for the differences in affinities of the quinoxaline ligands at 5-HT_3_A and 5-HT_3_AB receptors. As discussed below, several of the identified residues are known to interact with a range of 5-HT_3_ receptor ligands (Figures 2 and 3).^4^ Some of these are the same in both A+A− and A+B− binding sites and are unlikely to be responsible for differences in affinity, while others are different and may provide possible explanations for the varied ligand affinities at the two receptor subtypes.

Studies of the 5-HT_3_A receptor have identified an aromatic binding cavity formed by residues Trp 90 (loop D), Trp 183 (loop B) and Tyr 234 (loop C), mutagenesis of which effects both 5-HT activation and the binding of 5-HT_3_ receptor-competitive antagonists.^13^ Our homology models predict that both A+A− and A+B− binding sites contain these residues, providing an aromatic environment to accommodate the positively charged moiety that is a well-known pharmacophore feature of 5-HT_3_ ligands.^14^ Another pharmacophore feature, a hydrogen bond acceptor (HBA), is observed in both models as an interaction between the carbonyl oxygen atom of tropisetron and wat_2_ from a water network that has been observed in AChBP crystal structures; water molecules in this network are also stabilized by interactions with the backbone of the protein and the side chain of Tyr 234. In both binding sites, the positively charged moieties of tropisetron are also stabilized by ionic interactions with Glu 129 (loop A), and by a hydrogen bonding interaction between the protonated nitrogen atom and the carbonyl backbone of Trp 183. Mutagenesis studies have shown that both Glu 129 and Trp 183 are essential for 5-HT function and granisetron binding.^13^

Because the principle faces of both A+A− and A+B− binding sites are identical, we must look toward the A− and B− interfaces for differences between the two binding sites. Of the differing residues, A_Ile 71_/B_Phe 71_, A_Arg 92_/B_Gln 92_ and A_Gln 151_/B_Glu 151_ are closest to tropisetron, and might also be expected to interact with the structurally related quinoxaline ligands described here. A_Ile 71_/B_Phe 71_ lies in the β1-strand, close to binding loop A. However, A_Ile 71_ mutations to Ala and Leu had no effect on granisetron binding affinity, suggesting the residue at this location does not affect ligand binding.^13^ Conversely substitution of A_Arg 92_ changed the affinities of several 5-HT_3_ ligands, including 5-HT and granisetron, and we have previously speculated that a cation–π interaction could exist between A_Arg 92_ and the aromatic parts of (iso)quinolines and quinazolines, as it does with granisetron.^10^, ^15^ As this type of interaction would be absent in an A+B− site (as Gln is a neutral residue), quinoxalines might adopt quite a distinct orientation in this binding pocket. In support of this speculation, we have previously shown that introduction of a Cys substitution at this location has no effect on 5-HT or granisetron, but eliminates the allosteric effects of VUF10166 in heteromeric receptors.^7^ Similarly, the change in charge at location 151 (A_Gln 151_/B_Glu 151_) could have a significant effect on ligand binding properties. Although the effect of this residue has not yet been studied, mutation of the closely located B_Tyr 153_ residue also eliminates the allosteric effects of VUF10166, showing that this region influences binding at the B interface.^7^ For VUF10166, the effects of these B-substitutions are known to alter both the binding properties and the functional response, but for the other quinoxalines studied here, it has yet to be determined whether the differing binding affinities also translate into functional changes.

## Conclusions

In summary, most quinoxaline compounds examined here show no difference in their affinities at 5-HT_3_A and 5-HT_3_AB receptors, and we suggest that these compounds may only bind to the A+A binding site that is found in both receptor types, consistent with all other 5-HT_3_ receptor-competitive ligands.^10^, ^16^ Some, however, show significant differences and thus may bind to the A+B− interface as has previously been shown for VUF10166.^10^ These novel ligands could be valuable in both experimental and computer-aided drug design, with potential for the development of novel therapeutic agents.

## Experimental Section

**Chemistry**: Chemicals and solvents were purchased from Sigma–Aldrich and used as received. Unless indicated otherwise, all reactions were carried out under an inert atmosphere of dry N_2_. TLC analyses were performed with Merck F254 alumina silica plates using UV visualization or staining. Column purifications were carried out automatically using the Biotage equipment. All HRMS spectra were recorded on Bruker microTOF mass spectrometer using ESI in positive ion mode. ^1^H NMR spectra were recorded on a Bruker 250 (250 MHz) or a Bruker 500 (500 MHz) spectrometer. Data are reported as follows: chemical shift, integration, multiplicity (s=singlet, d=doublet, t=triplet, br=broad, m=multiplet), and coupling constants (Hz). Chemical shifts are reported in ppm with the natural abundance of deuterium in the solvent as the internal reference (CHCl_3_ in CDCl_3_: *δ*=7.26 ppm and CH_3_OH in CH_3_OD: *δ*=3.31 ppm, (CH_3_)_2_SO in (CD_3_)_2_SO: *δ*=2.50 ppm). ^13^C NMR spectra were recorded on a Bruker 500 (126 MHz) spectrometer with complete proton decoupling. Chemical shifts are reported in ppm with the solvent resonance resulting from incomplete deuteration as the internal reference (CDCl_3_: *δ*=77.16 ppm, CH_3_OD: *δ*=49.00 ppm, (CD_3_)_2_SO: *δ*=39.52 ppm). Systematic names for molecules according to IUPAC rules were generated using the ChemDraw AutoNom program. Purity was determined using a Shimadzu HPLC/MS workstation with a LC-20AD pump system, SPD-M20A diode array detection, and an LCMS-2010 EV mass spectrometer. An Xbridge C_18_ 5 μm column (100 mm×4.6 mm) was used. Compound purities were calculated as the percentage peak area of the analyzed compound by UV detection at 230 nm. Solvents used were as follows: solvent B=CH_3_CN 0.1 % formic acid; solvent A=H_2_O 0.1 %. The analysis was conducted using a flow rate of 1.0 mL min^−1^, starting at 5 % B with a linear gradient to 90 % B in 4.5 min, then 1.5 min at 90 % B with a linear gradient to 5 % B in 0.5 min, and then 1.5 min at 5 % B, with a total run time of 8 min. Compounds **24**–**34** were synthesized by our group as described by Smits et al.^9^

**3-Ethyl-3,4-dihydroquinoxalin-2(1*H*)-one (4)**: Benzene-1,2-diamine (**2**) (1.07 g, 28.4 mmol) and 2-oxobutanoic acid (2.90 g, 28.4 mmol) were dissolved in 50 mL CH_3_OH, and the resulting solution was stirred overnight at room temperature. The resulting precipitate was collected via filtration over a Büchner funnel. The precipitate was washed with cold CH_3_OH and dried in a vacuum oven to yield 2.25 g (12.9 mmol, 46 %) of **4** as an off-white solid: ^1^H NMR (500 MHz, CDCl_3_) *δ*=11.25 (s, 1 H), 7.84 (d, *J*=8.1 Hz, 1 H), 7.52–7.45 (m, 1 H), 7.36–7.31 (m, 1 H), 7.28 (d, *J*=7.8 Hz, 1 H), 3.01 (q, *J*=7.4 Hz, 2 H), 1.38 ppm (t, *J*=7.4 Hz, 3 H); ^13^C NMR (126 MHz, CDCl_3_) *δ*=162.54, 156.24, 132.91, 130.97, 129.57, 128.85, 124.09, 115.31, 26.85, 10.85 ppm.

**6,7-Dichloro-3,4-dihydroquinoxalin-2(1*H*)-one (5)**: 4,5-dichlorobenzene-1,2-diamine (**3**) (842 mg, 4.76 mmol) and 2-oxoacetic acid (715 mg, 4.83 mmol) were dissolved in CH_3_OH (50 mL) and stirred at room temperature for 30 min. The mixture was concentrated under reduced pressure, H_2_O was added, and the resulting mixture was extracted with EtOAc. The combined organic phases were dried over MgSO_4_ and concentrated under reduced pressure to yield 133 mg of **5** (0.62 mmol, 13 %) as a dark-brown solid: ^1^H NMR (250 MHz, DMSO) *δ*=8.19 (s, 1 H), 8.04 (s, 1 H), 7.45 ppm (s, 1 H).

**2-Ethyl-3-(4-methylpiperazin-1-yl)quinoxaline (6)**: A solution of **4** (1.64 g, 9.39 mmol) in phosphoryl trichloride (100 mL) was stirred at 100 °C for 1 h. The reaction mixture was then concentrated under reduced pressure. H_2_O was added to the remaining solid, then the mixture was extracted with CH_2_Cl_2_. The organic layers were combined, dried over Na_2_SO_4_, and concentrated under reduced pressure to yield 1.59 g (8.24 mmol, 88 %) of 2-chloro-3-ethylquinoxaline as a dark-pink solid: ^1^H NMR (250 MHz, CDCl_3_) *δ*=8.10–8.03 (m, 1 H), 8.02–7.95 (m, 1 H), 7.79–7.67 (m, 2 H), 3.17 (q, *J*=7.5 Hz, 2 H), 1.44 ppm (t, *J*=7.5 Hz, 3 H). Next, 2-chloro-3-ethylquinoxaline (555 mg, 2.88 mmol) was dissolved in *N*-methylpiperazine (2 mL), and the resulting solution was heated at 120 °C for 15 min using microwave (mw) radiation. After cooling to room temperature, excess *N*-methylpiperazine was removed under reduced pressure, and the product was purified over SiO_2_ (EtOAc/Et_3_N, 96:4, *v*/*v*) to yield 566 mg of **6** (2.21 mmol, 77 %) as a yellow solid: ^1^H NMR (250 MHz, CDCl_3_) *δ*=7.95–7.87 (m, 1 H), 7.85–7.78 (m, 1 H), 7.61–7.45 (m, 2 H), 3.43–3.31 (m, 4 H), 2.97 (q, *J*=7.4 Hz, 2 H), 2.68–2.58 (m, 4 H), 2.38 (s, 3 H), 1.41 ppm (t, *J*=7.4 Hz, 3 H); ^13^C NMR (126 MHz, CDCl_3_) *δ*=155.74, 154.15, 139.92, 138.88, 128.68, 128.01, 127.27, 126.57, 55.04, 49.67, 46.24, 27.70, 12.64 ppm; LCMS: *t*_R_=2.82 min, purity 95 %, [*M*+H]^+^ 257.00; HRMS *m*/*z*: [*M*+H]^+^ calcd for C_15_H_21_N_4_: 257.1761, found: 257.1763.

**6,7-Dichloro-2-(4-methylpiperazin-1-yl)quinoxaline (7)**: A solution of **5** (133 mg, 0.62 mmol) in phosphoryl trichloride (50 mL) was stirred at 100 °C for 1 h. The reaction mixture was concentrated under reduced pressure, H_2_O was added to the remaining solid this, and the mixture was extracted with CH_2_Cl_2_. The organic layers were combined, dried over Na_2_SO_4_, and concentrated under reduced pressure to yield 66 mg (0.28 mmol, 46 %) of 2,6,7-trichloroquinoxaline: ^1^H NMR (250 MHz, CDCl_3_) *δ*=8.77 (s, 1 H), 8.25 (s, 1 H), 8.15 ppm (s, 1 H). Then, 2,6,7-trichloroquinoxaline (66 mg, 0.28 mmol) was dissolved in EtOAc (2 mL), *N*-methylpiperazine (0.1 mL, 0.90 mmol) was added, and the resulting solution was heated at 160 °C for 1 h using microwave radiation. After cooling to room temperature, EtOAc and excess *N*-methylpiperazine were removed under reduced pressure, and the product was purified over SiO_2_ (EtOAc/Et_3_N, 96:4, *v*/*v*) to yield 36 mg of **7** (0.12 mmol, 43 %) as a light-brown solid: ^1^H NMR (250 MHz, CDCl_3_) *δ*=8.55 (s, 1 H), 7.96 (s, 1 H), 7.77 (s, 1 H), 3.85–3.79 (m, 4 H), 2.59–2.52 (m, 4 H), 2.37 ppm (s, 3 H); ^13^C NMR (126 MHz, CDCl_3_) *δ*=152.87, 142.50, 139.15, 136.67, 134.50, 131.10, 128.26, 127.62, 54.60, 48.80, 46.07 ppm; LCMS: *t*_R_=3.39 min, purity >99 %, [*M*+H]^+^ 296.90; HRMS *m*/*z*: [*M*+H]^+^ calcd for C_13_H_15_Cl_2_N_4_: 297.0668, found: 297.0662.

**3-(4-Methylpiperazin-1-yl)quinoxalin-2-amine (10)**: 2,3-Dichloroquinoxaline (**8**) (1.99 g, 10.0 mmol) was dissolved in a 2 m NH_3_ solution in EtOH (5.5 mL) and heated in the microwave at 100 °C for 2 h. The solvent was then removed under reduced pressure, and the residue was purified over SiO_2_ (CH_2_Cl_2_/EtOAc, 100:0 to 60:40, *v*/*v*) to give 300 mg (1.67 mmol, 17 %) of 3-chloroquinoxalin-2-amine. Next, 3-chloroquinoxalin-2-amine (150 mg, 0.84 mmol) and *N*-methylpiperazine (1.0 mL, 9.02 mmol) were dissolved in THF (4 mL). The resulting mixture was heated under microwave conditions at 150 °C for 40 min, quenched with H_2_O, and extracted with EtOAc. The organic layers were combined, dried (Na_2_SO_4_), and concentrated under reduced pressure. The product was crystallized from EtOAc to give 100 mg (0.41 mmol, 49 %) of **10** as a dark-yellow solid: ^1^H NMR (250 MHz, CDCl_3_) *δ*=7.76 (d, *J*=7.8 Hz, 1 H), 7.60 (d, *J*=7.8 Hz, 1 H), 7.50–7.33 (m, 2 H), 4.98 (s, 2 H), 3.56–3.27 (m, 4 H), 2.76–2.51 (m, 4 H), 2.46–2.29 ppm (m, 3 H); ^13^C NMR (126 MHz, CDCl_3_) *δ*=148.39, 147.69, 138.94, 137.41, 127.34, 127.26, 125.25, 125.08, 55.07, 48.54, 46.24 ppm; LCMS: *t*_R_=2.25 min, purity >99 %, [*M*+H]^+^ 244.00; HRMS *m*/*z*: [*M*+H]^+^ calcd for C_13_H_18_N_5_: 244.1557, found: 224.1552.

**2,6,7-Trichloro-3-(4-methylpiperazin-1-yl)quinoxaline (11)**: 2,3,6,7-Tetrachloroquinoxaline (**9**) (1,23 g, 4.58 mmol) was dissolved in THF (50 mL). *N*-methylpiperazine (0.58 mL, 4.58 mmol) and triethylamine (0.65 mL, 4.66 mmol) were added, and the mixture was stirred at 80 °C for 96 h, quenched with H_2_O, and extracted with EtOAc. The organic layers were combined, dried over MgSO_4_, and concentrated under reduced pressure to yield 1.02 g (3.08 mmol, 67 %) of **11** as a light-brown solid: ^1^H NMR (250 MHz, CDCl_3_) *δ*=7.95 (s, 1 H), 7.92 (s, 1 H), 3.68–3.59 (m, 4 H), 2.66–2.58 (m, 4 H), 2.38 ppm (s, 3 H); ^13^C NMR (126 MHz, CDCl_3_) *δ*=152.87, 142.50, 139.15, 136.67, 134.50, 131.10, 128.26, 127.62, 54.60, 48.80, 46.07 ppm; LCMS: *t*_R_=3.64 min, purity >99 %, [*M*+H]^+^ 330.85; HRMS *m*/*z*: [*M*+H]^+^ calcd for C_13_H_14_Cl_3_N_4_: 331.0279, found: 331.0271.

**2,3-Dibromoquinoxaline (13)**: Quinoxaline-2,3-diol (**12**) (2.96 g, 18.3 mmol) and pentabromophosphorane (17.06 g, 39.6 mmol) were suspended in toluene (200 mL) and heated at 160 °C for 3 h. After cooling to room temperature, ice water (200 mL) was added to the solution, and the mixture was stirred vigorously for 30 min. The mixture was extracted with toluene, washed with 1 n NaOH (100 mL), dried over MgSO_4_, filtered, and concentrated under vacuum. The crude product was purified over SiO_2_ (CH_2_Cl_2_/*n*-heptanes, 1:2, *v*/*v*) to give 505 mg (12.7 mmol, 70 %) of **13** as a beige solid: ^1^H NMR (250 MHz, CDCl_3_) *δ*=8.09–8.00 (m, 2 H), 7.86–7.77 ppm (m, 2 H).

**2-Bromo-3-(4-methylpiperazin-1-yl)quinoxaline (14)**: 2,3-Dibromoquinoxaline (**13**) (505 mg, 1.75 mmol), *N*-methylpiperazine (176 mg, 1.75 mmol), and Et_3_N (177 mg, 1.75 mmol) were dissolved in toluene (50 mL). The solution was stirred at 160 °C for 6 h. After cooling to room temperature, H_2_O was added, and the emulsion was extracted with toluene. The combined organic extracts were dried over MgSO_4_ and concentrated under vacuum. The crude product was purified over SiO_2_ (EtOAc/Et_3_N, 98:2, *v*/*v*) to give 377 mg (1.23 mmol, 70 %) of **14** as a beige solid: ^1^H NMR (250 MHz, CDCl_3_) *δ*=7.90 (dd, *J*=8.3, 1.2 Hz, 1 H), 7.83 (dd, *J*=8.3, 1.1 Hz, 1 H), 7.70–7.61 (m, 1 H), 7.58–7.48 (m, 1 H), 3.63–3.55 (m, 4 H), 2.69–2.61 (m, 4 H), 2.39 ppm (s, 3 H); ^13^C NMR (126 MHz, CDCl_3_) *δ*=153.60, 140.03, 139.18, 134.98, 130.25, 127.86, 127.40, 127.19, 54.70, 49.48, 46.18 ppm; LCMS: *t*_R_=2.69 min, purity >99 %, [*M*+H]^+^ 306.90; HRMS *m*/*z*: [*M*+H]^+^ calcd for C_13_H_16_BrN_4_: 307.0553, found: 307.0552.

**2-Chloro-3-(4-methyl-1,4-diazepan-1-yl)quinoxaline (16)**: 2,3-Dichloroquinoxaline (**8**) (1.00 g, 5.0 mmol), *N*-methyl-1,4-diazepane (0.86 mL, 7.50 mmol), and Et_3_N (0.70 mL, 5.00 mmol) were dissolved in toluene (50 mL). The solution was stirred overnight at reflux. After cooling to room temperature, H_2_O was added, and the resulting mixture was extracted with toluene, dried over MgSO_4_, and concentrated under vacuum. The crude was purified over SiO_2_ (EtOAc/Et_3_N, 99:1, *v*/*v*) to give 1.01 g (3.65 mmol, 73 %) of **16** as a yellow oil: ^1^H NMR (500 MHz, CDCl_3_) *δ*=7.87–7.76 (m, 1 H), 7.76–7.69 (m, 1 H), 7.58 (ddd, *J*=8.4, 7.0, 1.4 Hz, 1 H), 7.44 (ddd, *J*=8.3 7.0, 1.4 Hz, 1 H), 3.90–3.86 (m, 2 H), 3.86–3.81 (m, 2 H), 2.90–2.83 (m, 2 H), 2.70–2.64 (m, 2 H), 2.42 (s, 3 H), 2.10 ppm (dt, *J*=7.0, 6.0 Hz, 2 H); ^13^C NMR (126 MHz, CDCl_3_) *δ*=151.91, 140.04, 139.18, 137.27, 130.04, 127.52, 126.40, 126.04, 58.57, 57.51, 50.61, 50.33, 46.93, 28.06 ppm; LCMS: *t*_R_=2.89 min, purity >99 %, [*M*+H]^+^ 277.10; HRMS *m*/*z*: [*M*+H]^+^ calcd for C_14_H_18_ClN_4_: 277.1215, found: 277.1210.

***tert*****-Butyl (1-(3-chloroquinoxalin-2-yl)pyrrolidin-3-yl)(methyl)carbamate (17)**: 2,3-dichloroquinoxaline (0.54 g, 2.71 mmol) was dissolved in DMF (30 mL). K_2_CO_3_ (0.37 g, 2.71 mmol) and *tert*-butyl methyl(pyrrolidin-3-yl)carbamate (0.49 g, 2.47 mmol) were added, and the mixture was stirred at 90 °C for 4 h. The mixture was cooled to room temperature, diluted with H_2_O, and extracted with Et_2_O. The combined organic layers were dried over MgSO_4_ and concentrated under reduced pressure to give 0.68 g of **17**, which was directly used in the synthesis of **18**.

**1-(3-Chloroquinoxalin-2-yl)-*N*-methylpyrrolidin-3-amine (18)**: Compound **17** (0.40 g) was dissolved in dioxane (10 mL) and stirred at room temperature. A 4 m solution of HCl in dioxane (20 mL) was added dropwise, and precipitation was observed. The resulting suspension was stirred overnight and subsequently filtered over a Büchner funnel, and the residue was washed with 1,4-dioxane. The residue was then dried under reduced pressure to yield 202 mg of **18** as a light-yellow solid (0.68 mmol, 61 %): ^1^H NMR (500 MHz, CDCl_3_) *δ*=7.87–7.74 (m, 2 H), 7.68 (ddd, *J*=8.4, 7.1, 1.4 Hz, 1 H), 7.58–7.46 (m, 1 H), 4.27–4.10 (m, 3 H), 4.10–3.92 (m, 2 H), 2.83 (s, 3 H), 2.61–2.45 (m, 1 H), 2.37–2.23 ppm (m, 1 H); ^13^C NMR (126 MHz, CDCl_3_) *δ*=147.42, 137.60, 136.72, 136.02, 131.08, 127.39, 126.50, 123.29, 57.92, 52.40, 48.69, 31.08, 27.60 ppm; LCMS: *t*_R_=2.90 min, purity 99 %, [*M*+H]^+^ 263.05; HRMS *m*/*z*: [*M*+H]^+^ calcd for C_13_H_16_ClN_4_: 263.1058, found: 263.1055.

**2-(4-Methyl-1,4-diazepan-1-yl)quinoxaline (19)**: 2-Chloroquinoxaline (**15**) (2.97 g, 18.0 mmol), *N*-methyl-1,4-diazepane (3.3 mL, 24.0 mmol), and Et_3_N (2.5 mL, 18.0 mmol) were dissolved in toluene (50 mL). The solution was stirred overnight at reflux. After cooling to room temperature, H_2_O was added, and the resulting mixture was extracted with toluene, dried over MgSO_4_, and concentrated under vacuum. The crude residue was purified over SiO_2_ (EtOAc/Et_3_N, 98:2, *v*/*v*) to give 3.12 g (12.9 mmol, 71 %) of **19** as an off-white solid: ^1^H NMR (250 MHz, CDCl_3_) *δ*=8.47 (s, 1 H), 7.86 (d, *J*=8.2 Hz, 1 H), 7.65 (d, *J*=8.4 Hz, 1 H), 7.59–7.49 (m, 1 H), 7.39–7.29 (m, 1 H), 4.02–3.93 (m, 2 H), 3.86 (t, *J*=6.3 Hz, 2 H), 2.77 (dd, *J*=5.7, 4.2 Hz, 2 H), 2.65–2.53 (m, 2 H), 2.39 (s, 3 H), 2.15–2.00 ppm (m, 2 H); ^13^C NMR (126 MHz, CDCl_3_) *δ*=151.59, 142.03, 136.46, 134.92, 129.96, 128.65, 126.33, 123.98, 58.18, 57.25, 46.73, 46.55, 46.24, 27.48 ppm; LCMS: *t*_R_=2.40 min, purity >99 %, [*M*+H]^+^ 243.00; HRMS *m*/*z*: [*M*+H]^+^ calcd for C_14_H_19_N_4_: 243.1604, found: 243.1608.

***tert*****-Butyl methyl(1-(quinoxalin-2-yl)pyrrolidin-3-yl)carbamate (20)**: 2-Chloroquinoxaline (1.79 g, 10.9 mmol) was dissolved in DMF (50 mL). K_2_CO_3_ (1.51 g, 10.9 mmol) and *tert*-butyl methyl(pyrrolidin-3-yl)carbamate (2.00 g, 10.0 mmol) were added, and the mixture was stirred at 90 °C for 6 h. The mixture was cooled to room temperature, diluted with H_2_O, and extracted with Et_2_O. The combined organic layers were dried over MgSO_4_ and concentrated under reduced pressure to give 3.28 g of **20**, which was directly used in the synthesis of **21**.

***N*****-Methyl-1-(quinoxalin-2-yl)pyrrolidin-3-amine (21)**: Compound **20** (2.95 g) was dissolved in dioxane (20 mL) and stirred at room temperature. A solution of 4 m HCl in dioxane (10 mL) was added dropwise, and precipitation was observed. The suspension was stirred overnight and filtered over a Büchner funnel. The residue was washed with 1,4-dioxane and dried under vacuum to yield 1.33 g of **21** as a beige solid (5.02 mmol, 56 %): ^1^H NMR (500 MHz, DMSO) *δ*=9.69–9.52 (m, 2 H), 8.58 (s, 1 H), 7.89–7.82 (m, 1 H), 7.72 (d, *J*=8.2 Hz, 1 H), 7.67–7.58 (m, 1 H), 7.45–7.37 (m, 1 H), 4.00–3.85 (m, 4 H), 3.78–3.69 (m, 1 H), 2.65–2.58 (m, 3 H), 2.46–2.29 ppm (m, 2 H); ^13^C NMR (126 MHz, DMSO) *δ*=149.63, 140.28, 138.07, 136.38, 130.79, 129.12, 125.36, 124.68, 57.49, 49.25, 45.20, 31.42, 27.86 ppm; LCMS: *t*_R_=2.41 min, purity 97 %, [*M*+H]^+^ 229.10; HRMS *m*/*z*: [*M*+H]^+^ calcd for C_13_H_17_N_4_: 229.1448, found: 229.1454.

**3,6-Dichloro-2-(4-methylpiperazin-1-yl)quinoxaline (23)**: DiPEA (0.38 mL, 2.15 mmol) and POCl_3_ (2.00 mL, 21.5 mmol) were added to a solution of 7-chloro-3-(4-methylpiperazin-1-yl)quinoxalin-2(1*H*)-one (**22**) (300 mg, 1.08 mmol) in toluene (20 mL). The resulting mixture was stirred at reflux for 20 h, after which the mixture was concentrated under reduced pressure. H_2_O (50 mL) and 1 m NaOH_(aq)_ (10 mL) were added to the crude product, and the resulting mixture was extracted with CH_2_Cl_2_. The combined organic layers were washed with brine (50 mL), dried over NaSO_4_, and concentrated under reduced pressure. The crude product was purified over SiO_2_ (EtOAc/Et_3_N, 50:1, *v*/*v*) to give 270 mg of **23** (0.91 mmol, 84 %) as a yellow solid: ^1^H NMR (500 MHz, CDCl_3_) *δ*=7.85 (d, *J*=2.3 Hz, 1 H), 7.75 (d, *J*=8.9 Hz, 1 H), 7.57 (dd, *J*=8.9, 2.3 Hz, 1 H), 3.69–3.54 (m, 4 H), 2.72–2.56 (m, 4 H), 2.38 ppm (s, 3 H); ^13^C NMR (126 MHz, CDCl_3_) *δ*=152.58, 142.61, 138.75, 138.30, 132.59, 130.91, 128.14, 126.71, 54.70, 48.94, 46.15 ppm; LCMS: *t*_R_=3.40 min, purity <99 %, [*M*+H]^+^ 196.90; HRMS *m*/*z*: [*M*+H]^+^ calcd for C_13_H_15_Cl_2_N_4_: 297.0668, found: 297.0671.

*Radioligand binding*: This was carried out as previously described.^6^, ^7^, ^10^ Briefly, HEK293 cells expressing either 5-HT_3_A or 5-HT_3_AB receptors were scraped into 1 mL of ice-cold HEPES buffer (10 mm, pH 7.4) and frozen. After thawing, they were washed with HEPES buffer and homogenized using a fine-bore syringe. For competition binding experiments, 50 μL of cell membranes were incubated in 0.5 mL HEPES buffer containing a final concentration of 0.7 nm [^3^H]granisetron (∼*K*_d_), both with and without the test compound. To ensure that there were no changes in the *K*_d_ of [^3^H]granisetron, which could influence the *K*_i_ values of competing ligands, saturation binding curves were run in parallel with competition studies. Competition binding experiments using ten concentrations of ligands were performed on at least three separate plates of cells. Nonspecific binding was determined using 1 mm quipazine. Reactions were incubated for at least 24 h at 4 °C to allow compounds with slow kinetics to equilibrate. Experiments on 5-HT_3_A and 5-HT_3_AB receptors were run in parallel. Incubations were terminated by vacuum filtration using a Brandel cell harvester (Alpha Biotech Ltd., London, UK) onto GF/B filters pre-soaked in 0.3 % polyethyleneimine. Radioactivity was determined by scintillation counting. Data were fit according to Equation (1):


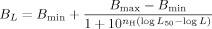
(1)

in which *L* is the concentration of ligand present, *B_L_* is the binding in the presence of ligand concentration *L*, *B*_min_ is the binding when *L*=0, *B*_max_ is the binding when *L*=∞, *L*_50_ is the concentration of *L w*hich gives a binding equal to (*B*_max_+*B*_min_)/2, and *n*_H_ is the Hill coefficient. *K*_i_ values were estimated from IC_50_ values using the Cheng–Prusoff equation^17^ *K*_i_=IC_50_/(1+[*L*]/*K*_d_), for which *K*_i_ is the equilibrium dissociation constant for binding of the unlabeled antagonist, IC_50_ is the concentration of antagonist that blocks half the specific binding, [*L*] is the free concentration of radioligand, and *K*_d_ is the equilibrium dissociation constant of the radioligand.

*Homology modeling*: Construction of the homomeric 5-HT_3_A receptor binding site model has been previously described.^18^ Using the same approach, a model of the 5-HT_3_AB receptor binding site was constructed by homology modeling using MOE (version 2010.10, Chemical Computing Group, Montreal). The sequence of the human 5-HT_3_AB gene (O95264) was aligned with the sequence of the 5-HT_3_A gene (P46098) using the “Protein Align” option in MOE (standard settings) and was adjusted manually. The final sequence alignment is shown in Figure 2. The 5-HT_3_A receptor homology model was selected to serve as the template. Structural waters located in the binding pocket of the original crystal structure (PDB code: 2WNC)^19^ form a conserved protein–ligand hydrogen bond interaction network in several other AChBP crystals (e.g., 2BYR, 2PGZ, 2BYS, 2XYT) and were included in the 5-HT_3_AB receptor model. The template backbone, the ligand, and the water molecules were fixed, and ten receptor models were constructed based on the template backbone. During this construction, the ligand and waters of the original co-crystal structure were considered as an additional restraint using the “Environment” option within MOE. The structural quality of the models was checked using the evaluation modules in MOE; protein geometry of receptor atoms was evaluated for bond lengths, bond angles, atom clashes, and contact energies. Ramachandran plots were used to check the Phi and Psi angles of all residues. The best model was selected for further refinement, hydrogen atoms were added, partial atomic charges were calculated, and the protein was minimized around the fixed ligand and static water molecules using the Amber99 force field in MOE.
